# Liver fibrotic burden across the spectrum of hypothyroidism

**DOI:** 10.1007/s00535-024-02184-x

**Published:** 2024-11-27

**Authors:** Tingting Du, Yuchai Huang, Yongman Lv, Gang Yuan

**Affiliations:** 1 Department of Endocrinology, Tongji Hospital, Tongji Medical College, Huazhong University of Science and Technology, Wuhan, 430030 China; 2 Branch of National Clinical Research Center for Metabolic Diseases, Wuhan, China; 3 Department of Health Management Centre, Tongji Hospital, Tongji Medical College, Huazhong University of Science and Technology, Wuhan, Hubei China; 4 Department of Nephrology, Tongji Hospital, Tongji Medical College, Huazhong University of Science and Technology, Wuhan, Hubei China

**Keywords:** Metabolic dysfunction-associated steatotic liver disease, Hypothyroidism, Significant fibrosis, Advanced fibrosis, Liver stiffness

## Abstract

**Background:**

Data regarding the prevalence of hepatic fibrotic burden across the spectrum of hypothyroidism are scarce. Hence, we aimed to evaluate the prevalence of liver fibrotic burden across the spectrum of hypothyroidism.

**Methods:**

30,091 individuals who attended a Health Management Centre between 2019 and 2021 were cross-sectionally analyzed. Participants were categorized as having strict-normal thyroid function, low-normal thyroid function, subclinical hypothyroidism, and overt hypothyroidism. Hepatic fibrosis was assessed by vibration-controlled transient elastography (VCTE). Significant and advanced fibrosis were defined as liver stiffness measurement in VCTE of 8.1–9.6 and 9.7–13.5 kPa, respectively.

**Results:**

Among both men and women, low-normal thyroid function group, subclinical hypothyroidism group, and overt hypothyroidism group all have more liver fibrosis present, including mild fibrosis, significant fibrosis, advanced fibrosis, and cirrhosis, than the strict-normal thyroid function group. The low-normal thyroid function group have the similar liver fibrotic burden to the subclinical hypothyroidism group. The highest liver fibrotic burden was noted in the overt hypothyroidism group. Both significant and advanced liver fibrosis were significantly associated with low-normal thyroid function, subclinical hypothyroidism, and overt hypothyroidism in both men and women.

**Conclusions:**

Liver fibrotic burden are highly prevalent in subjects with overt hypothyroidism. Moreover, fibrotic burden increased across the spectrum of hypothyroidism even within the low normal thyroid function. These results suggested that screening for liver fibrosis in patients with hypothyroidism is necessary.

## Introduction

Metabolic dysfunction-associated steatotic liver disease (MASLD), formerly known as non-alcoholic fatty liver disease (NAFLD), currently affecting more than 30% of the global population [1]. Evidence supports a role for hypothyroidism, even the low-normal thyroid function (serum thyroid stimulating hormone [TSH] concentrations at the upper end of the normal range), as the driver of the progression of MASLD to its severe forms [2, 3]. Further, the spectrum of hypothyroidism has been associated with an increased risk of cardiovascular and all-cause mortality, which is already increased in MASLD [4, 5]. These findings emphasize the significant influence of hypothyroidism on clinical progression of MASLD, even within the euthyroid reference range.

Although the prevalence of MASLD across the spectrum of hypothyroidism have been evaluated [6], the prevalence of liver fibrotic burden across the full spectrum of hypothyroidism has not been studied. Further, all studies that evaluated liver fibrosis in patients with hypothyroidism used serum biomarkers [2, 3], which may have suboptimal sensitivity for identifying stages of fibrosis. There are currently no data available on liver fibrotic burden assessed by imaging such as vibration-controlled transient elastography (VCTE) across the spectrum of hypothyroidism. VCTE has been shown to be excellent for the quantification of liver stiffness in validation studies against liver biopsy [7]. Considering that liver fibrosis is the major prognostic factor in MASLD [8], and that low-normal thyroid function and subclinical hypothyroidism are highly prevalent in the general adult population [9, 10], it is essential to screen hepatic fibrotic burden across the spectrum of hypothyroidism. Hence, we aimed to examine the prevalence of liver fibrosis assessed by VCTE across the spectrum of hypothyroidism. Moreover, since different metabolic traits present significant differences in the degree of liver fibrosis [11], we further evaluated the fibrotic burden by metabolic traits across the spectrum of hypothyroidism.

## Methods

### Study population

Data for this study were drawn from the Health Management Centre of Tongji Hospital, Huazhong University of Science and Technology (Wuhan, China). This Health Management Center is the largest healthcare center in Central China. It provides health checks for the entire 13 districts in Wuhan. Nowadays, people living in residential communities generally attend the center for annual health checks. Most participants were employees of institutions or corporations in Wuhan city and their health checks are funded by employment benefits. Other participants like the self-employed and farmers would pay for the health checkup themselves. There are four optional medical checkup packages provided by the Health Management Centre. People chose their medical checkup packages according to their personal wishes. The present study included a cohort of Chinese men and women aged ≥ 18 years who choose medical checkup packages containing a comprehensive health examination, thyroid function tests, and VCTE examination at the Health Management Center between January 2019 and December 2021. For those who attended at least two check-ups, only the most recent medical evaluation documentation was kept for the analyses. The survey procedures were reviewed and approved by the ethics committee of Tongji Hospital, Huazhong University of Science and Technology in accordance with the ethical standards laid down in the 1964 Declaration of Helsinki and its later amendments. According to the Private Information Protection Law, personal identification information was safeguarded by the Computer Center. Because we only retrospectively accessed a de-identified database, informed consent requirement was exempted by the institutional review board. All research was conducted in accordance with the ethical standards laid down in the 1964 Declaration of Helsinki and its later amendments.

**Fig. 1:**
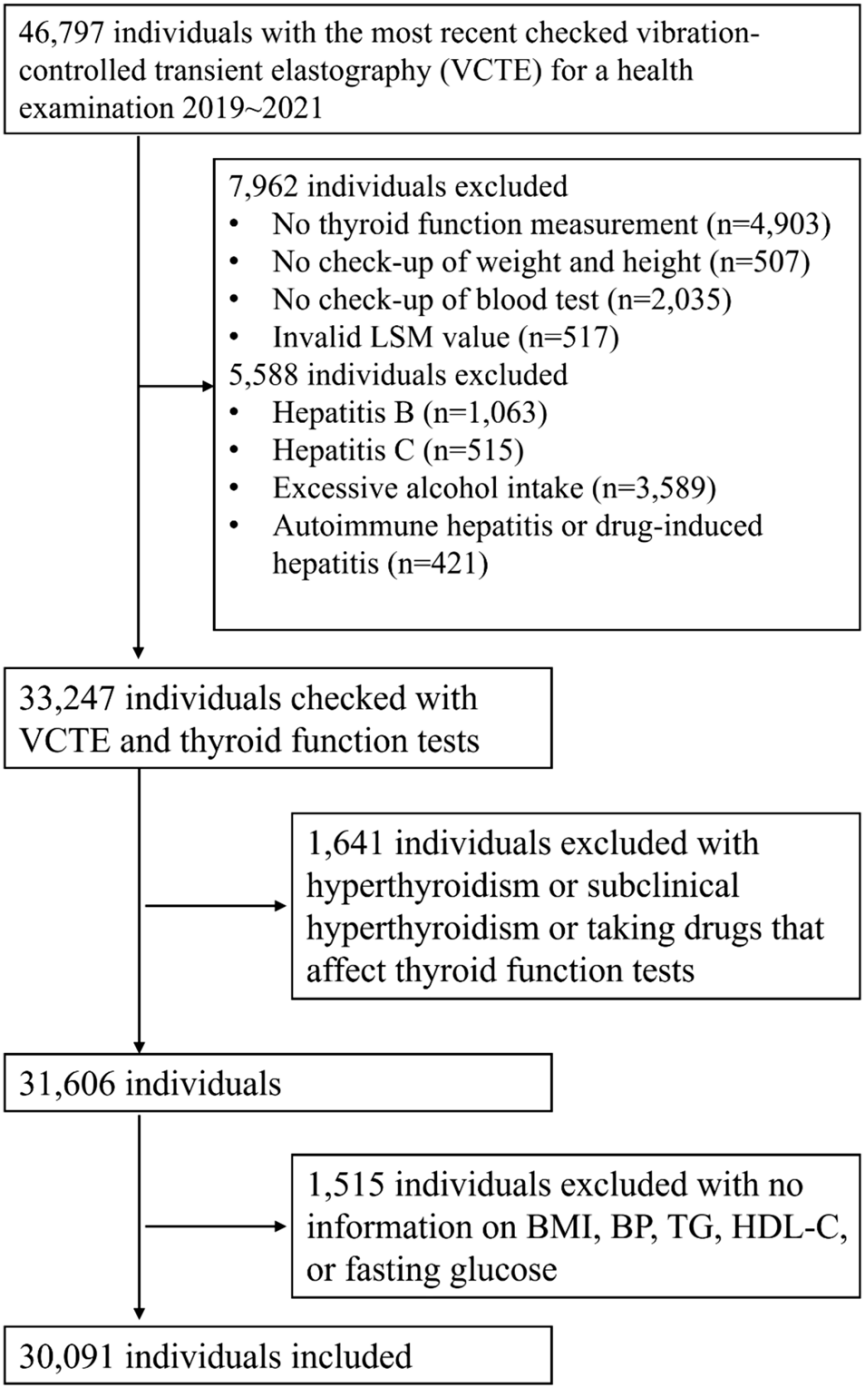
The flow diagram for the target population included in the study

All subjects were asked to complete a standard questionnaire that collected information on age, sex, alcohol consumption, histories of current and previous illness, and medication use. Information on medication use were shown in Supplementary Table 1. Exclusion criteria were an established history of hyperthyroidism, sub-hyperthyroidism, liver disease other than MASLD (i.e., hepatitis B or C, autoimmune hepatitis, history of excessive alcohol intake [> 30 g/day for men and > 20 g/day for women], Wilson disease, hereditary hemochromatosis, drug-induced hepatitis, etc.), taking thyroid medications (thyroid hormone or antithyroid drugs), on medications that might interfere with thyroid function, or with no information on liver stiffness measurement (LSM), thyroid function test, or cardiometabolic risk factors that used to define MASLD. The remaining available 30.091 participants were included in our data analysis. The flow diagram for the target population in the study was shown in Fig. 1.

### Anthropometric and biochemical measurements

Weight, height, and blood pressure (BP) were measured following standardized protocols from the World Health Organization (WHO). BMI was calculated as weight (in kilograms) divided by the square of height (in meters).

The Chinese version of alcohol use disorders identification test (AUDIT), with ten items was used to estimate drinking severity. The AUDIT is an international self-administered questionnaire used to detect non-serious alcohol-related problems, and it was validated for use in China. Total scores ranged from 0 to 40; scores of 1–7, 8–14, and ≥ 15 indicated low risk drinking, harmful drinking, and alcohol dependence, respectively.

A 12-h overnight fasting blood sample was collected from the antecubital vein. Biochemical measurements, including fasting plasma glucose (FPG), total cholesterol (TC), triglycerides (TG), low-density lipoprotein cholesterol (LDL-C), high-density lipoprotein cholesterol (HDL-C), alanine aminotransferase (ALT), and aspartate aminotransferase (AST) were measured with standard methods on a biochemistry autoanalyzer (Roche Cobas 8000 modular analyzer). HbA1c was measured using high performance liquid chromatography (D-10^™^; Bio‐Rad Laboratories, Hercules, CA, USA). TSH, free triiodothyronine (fT3) and thyroxine (fT4) were measured using the radioimmunology assay. The reference ranges for TSH were 0.27–4.2 μIU/ml, for fT3 3.1–6.8 pmol/L, and for fT4 12–22 pmol/L. All blood measurements were followed the same protocol.

### Abdominal ultrasound

Abdominal ultrasound was performed by certified sonographers using a high-resolution, real-time scanner. Generally, the ultrasonographic diagnosis of steatotic liver was based on the following criteria: [12] the brightness of liver parenchyma, presence of liver to kidney contrast, presence of deep beam attenuation, and vascular blurring.

### Vibration-controlled transient elastography

LSM and controlled attenuation parameter (CAP) by VCTE were measured using the FibroScan model 502 V2 Touch (Echosens, Paris, France) equipped with both medium (M) and extra-large (XL) probes. The M probe was used initially unless the machine recommended use of the XL probe. Participants were asked to fast for at least 8 h before the exam and were kept in a supine position throughout the examination and measurements were performed by scanning the right liver lobe through an intercostal space. The operator obtained at least ten measurements, and the device calculated the values of median LSM in kilopascals (kPa) together with the interquartile range (IQR) for each participant. Examinations were considered reliable if a minimum of ten valid LSM values were obtained, with an IQR/median less than 30%. Hepatic steatosis was measured using CAP in decibels per meter (dB/m) with median and IQR calculated for each participant.

### Definitions

#### Presence and severity of steatosis and fibrosis

Patients were considered to have CAP-defined steatosis if median CAP values ≥ 248 dB/m [13].

According to cutoffs from existing studies [7], presence of liver fibrosis was defined as follows: F1 (mild) if LSM in VCTE 7.0–8.0 kPa, F2 (significant fibrosis) if LSM 8.1–9.6 kPa, F3 (advanced fibrosis) if LSM 9.7–13.5 kPa, and F4 (cirrhosis) if LSM ≥ 13.6 kPa.

#### Thyroid function status

Strict-normal thyroid function was defined as TSH 0.27–2.4 mIU/L and fT3 and fT4 concentrations within the normal range according to data from our reference laboratory. Low-normal thyroid function was defined as TSH 2.5 to 4.2 mIU/L and euthyroid fT3 and T4 levels. Subclinical hypothyroidism was defined as TSH > 4.2 mIU/L and euthyroid fT3 and fT4 levels. Overt hypothyroidism was defined as TSH > 4.2 mIU/L with low fT3 or fT4 levels.

#### Definition of MASLD

Following the international expert consensus statement [14], MASLD was defined as ultrasonographic diagnosis of hepatic steatosis and fulfilled at least one of the following five cardiometabolic abnormalities (ultrasonographic definition): (1) BMI ≥ 23 kg/m^2^, (2) BP ≥ 130/85 mmHg or taking anti-hypertensive medications, (3) FPG ≥ 5.6 mmol/L, or HbA1c ≥ 5.7% or taking anti-diabetic medications, (4) TG ≥ 1.7 mmol/L or taking lipid-lowering medications, (5) HDL-C < 1.0/1.3 mmol/L for men/women.

MASLD was also defined as evidence of hepatic steatosis detected by CAP ≥ 248 dB/m in VCTE plus at least one of the cardiometabolic abnormalities mentioned above (CAP definition).

### Statistical analysis

All statistical analyses were conducted using SAS 9.4 (SAS Institute Inc., Cary, North Carolina). Continuous variables were presented as means (standard deviations, SDs). Logarithmic transformation was performed where needed. Categorical variables were presented as numbers (percentages). Differences in continuous variables between groups were tested with ANOVA. Bonferroni method was used for post hoc testing. Differences in categorical variables were tested with *χ*2 test. Trends in prevalence of MASLD and fibrotic burden across the spectrum of hypothyroidism were assessed by Cochran–Armitage trend testing. Logistic regression models were used to estimate the associations (odds ratios [ORs], with 95% confidence Intervals [CIs]) between thyroid function status and risk of significant or advanced fibrosis. All models were adjusted for age, sex, BMI, systolic BP, HbA1c, TC, LDL-C, ALT, total bilirubin, the presence of MASLD, anti-diabetic drugs, anti-hypertensive drugs, and lipid-lowering drugs. Significance was accepted at a two-tailed *P* < 0.05.

## Results

Low-normal thyroid function, and subclinical hypothyroidism were present in 23.3% and 7.1% of participants, respectively. Prevalence of ultrasonographic-defined and CAP-defined MASLD in the overall population was 39.2%, and 52.8%, respectively.

As shown in Table 1, age, systolic/diastolic BP, FPG, HbA1c, TC, ALT, AST, LSM, and TSH showed a progressive increase from the group with strict-normal thyroid function to the group with overt hypothyroidism (all *P* for trend < 0.05). This was also the case for the prevalence of cardiometabolic disorders (all *P* for trend < 0.05).

**Table 1:** Characteristics of the study participants across the spectrum of hypothyroidism

	Strict-normal thyroid function	Low-normal thyroid function	Subclinical hypothyroidism	Overt hypothyroidism	*P* for trend
*n*	20,193	7369	2249	280	
Men (%)	68.3	58.7	50.9	44.6	< 0.0001
Age (year)	45.0 ± 10.8	46.8 ± 11.2	49.3 ± 11.5	53.5 ± 9.9	< 0.0001
Body mass index (kg/m^2^)	25.2 ± 3.9	24.6 ± 3.6	24.5 ± 4.7	25.0 ± 3.4	0.9091
Systolic blood pressure (mmHg)	126.5 ± 17.4	127 ± 17.4	128.5 ± 18.5	133 ± 20.3	< 0.0001
Diastolic blood pressure (mmHg)	79.0 ± 12.0	79.1 ± 12.0	79.6 ± 12.2	81.1 ± 12.8	0.0061
Fasting plasma glucose (mmol/L)	5.4 ± 1.3	5.4 ± 1.4	5.4 ± 1.4	5.4 ± 0.9	0.9156
Alanine aminotransferase (IU/L)	23.8 ± 21.4	23.5 ± 19.6	22.9 ± 17.2	23.8 ± 23.9	0.1934
Aspartate aminotransferase (IU/L)	21.8 ± 11.2	22.5 ± 18.2	22.5 ± 10.3	23.7 ± 12.2	0.0013
HbA1c (%)	5.7 ± 0.7	5.7 ± 0.7	5.8 ± 0.7	5.8 ± 0.6	0.0041
Total cholesterol (mmol/L)	4.6 ± 0.9	4.7 ± 0.9	4.7 ± 0.9	4.8 ± 1.0	0.0002
Triglycerides (mmol/L)	1.6 ± 1.5	1.7 ± 1.6	1.6 ± 1.4	1.8 ± 1.6	0.0604
LDL-cholesterol (mmol/L)	2.9 ± 0.8	2.9 ± 0.8	3.0 ± 0.8	3.0 ± 0.9	0.0862
HDL-cholesterol (mmol/L)	1.2 ± 0.3	1.2 ± 0.3	1.3 ± 0.3	1.2 ± 0.3	0.9753
Controlled attenuation parameter (kPa)	252.7 ± 36.1	252.7 ± 36.8	251.1 ± 34.6	256.2 ± 36.3	0.1741
Uric acid (µmol/L)	360.7 ± 97.9	355.6 ± 102.0	348.5 ± 100.4	334.2 ± 95.3	< 0.0001
Creatinine (µmol/L)	75.8 ± 18.0	75.1 ± 18.8	75.4 ± 30.6	78.3 ± 75.0	0.0412
Thyroid stimulating hormone (mIU/L)	1.6 ± 0.5	3.1 ± 0.5	6.0 ± 3.1	16.3 ± 21.0	< 0.0001
Free triiodothyronine (pmol/L)	5.1 ± 0.6	5.0 ± 0.6	4.9 ± 0.6	3.9 ± 1.0	< 0.0001
Free thyroxine (pmol/L)	16.4 ± 2.0	15.9 ± 1.9	15.3 ± 2.0	8.4 ± 4.2	< 0.0001
Body mass index ≥ 23 kg/m^2^ (%)	66.2	65.7	65.3	70.7	0.6884
Systolic/diastolic blood pressure ≥ 130/85 mmHg (%)	29.6	31.1	34.0	42.5	< 0.0001
Triglycerides ≥ 1.7 mmol/l (%)	31.5	32.3	32.1	39.6	0.0366
HDL-cholesterol < 1.0/1.3 mmol/L in men/women (%)	32.1	35.7	36.1	41.4	< 0.0001
Fasting glucose ≥ 5.6 mmol/L or HbA1c ≥ 5.7% (%)	38.1	39.8	43.8	54.3	< 0.0001
One cardiometabolic disorder (%)	22.6	22.2	21.3	17.9	< 0.0001
Two cardiometabolic disorders (%)	23.1	22.3	23.9	25.7	
Three cardiometabolic disorders (%)	20.7	21.4	22.5	22.1	
Four cardiometabolic disorders (%)	12.8	13.3	14.6	21.4	
Five cardiometabolic disorders (%)	4.1	5.3	4.4	6.8	
Diabetes (%)	7.5	8.0	7.9	10.1	0.049

Data were presented as means (standard deviations, SDs) or percentages (%)

*P* for trend was analyzed using generalized linear model

FT3 and fT4 showed a progressive decrease (*P* for trend < 0.001).

**Fig. 2:**
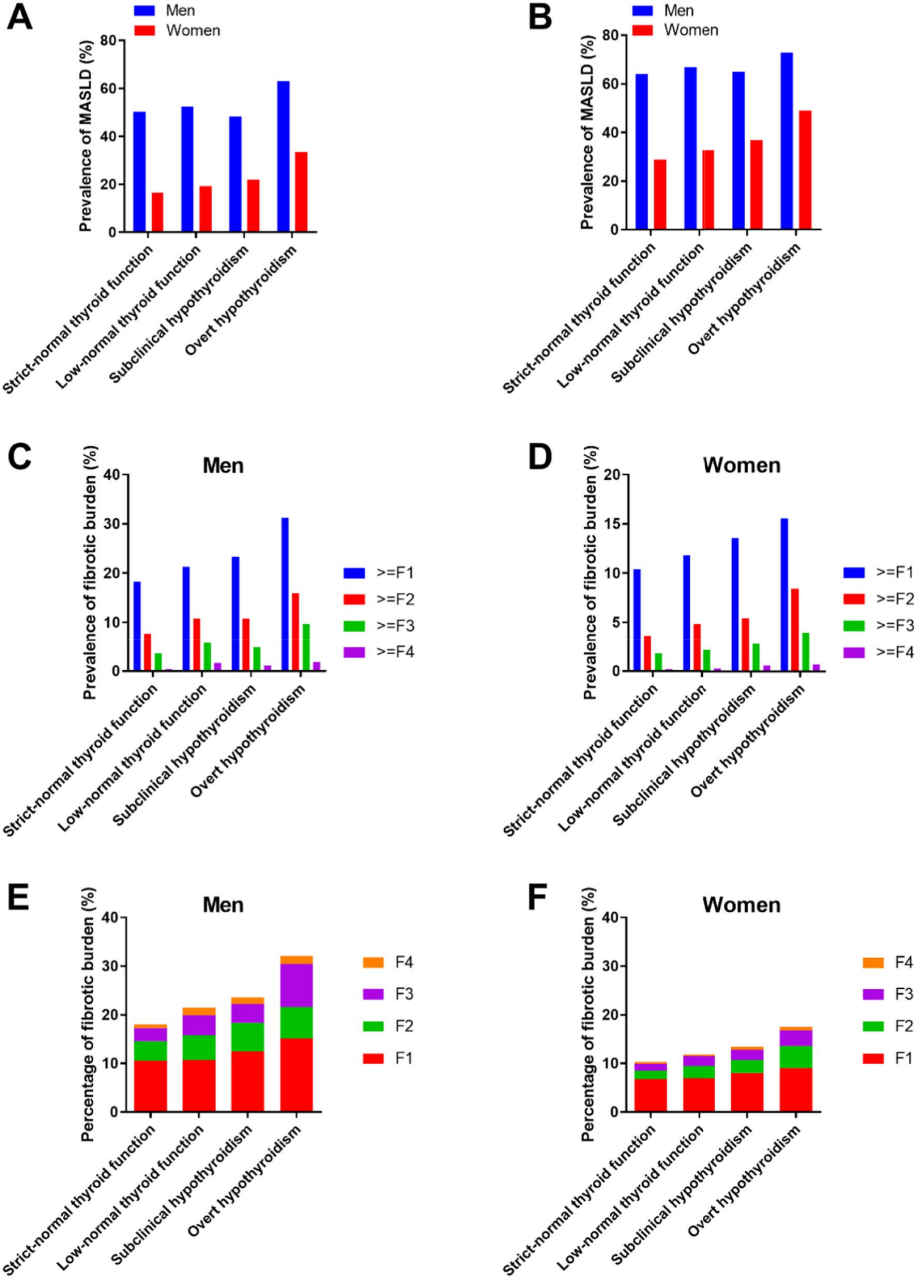
Metabolic dysfunction-associated steatotic liver disease (MASLD) and fibrotic burden across the spectrum of hypothyroidism in men and women. **A** Prevalence of ultrasonographic-defined MASLD across the spectrum of hypothyroidism in men and women; **B** Prevalence of controlled attenuation parameter-defined MASLD across the spectrum of hypothyroidism in men and women; **C** Prevalence of fibrotic stages across the spectrum of hypothyroidism in men; **D** Prevalence of fibrotic stages across the spectrum of hypothyroidism in women; **E** Prevalence of fibrotic burden in each hypothyroidism status in men. **F** Prevalence of fibrotic burden in each hypothyroidism status in women

Since accumulating evidence has shown sex disparities in the epidemiology, progression, and outcomes of hypothyroidism and MASLD [15, 16], we stratified the analyses by sex. In men, the MASLD prevalence increased from 50.3% in individuals with strict-normal thyroid function to 62.9% in patients with overt hypothyroidism using ultrasonographic definition (Fig. 2A), and from 63.9% to 72.8% in the same categories using CAP definition (*P* trend < 0.001, Fig. 2B). In women, the corresponding figures were 16.5% and 33.4%, respectively, using ultrasonographic definition (*P* trend < 0.001, Fig. 2A) and 28.9% and 49%, respectively, using CAP definition, respectively (*P* trend < 0.001, Fig. 2B).

Liver fibrosis stage across the spectrum of hypothyroidism is presented in Fig. 2C–F. Among both men and women, low-normal thyroid function group, subclinical hypothyroidism group, and overt hypothyroidism group all have more fibrosis present, including mild fibrosis, significant fibrosis, advanced fibrosis, and cirrhosis, than strict-normal thyroid function group (*P* trend < 0.001, Fig. 2C–F). The low-normal thyroid function group have the similar liver fibrotic burden to subclinical hypothyroidism group. The highest liver fibrotic burden was noted in the overt hypothyroidism group. The significant fibrosis and advanced fibrosis reached 16.0% and 9.6%, respectively, in patients with overt hypothyroidism in men (Fig. 2C). In women, the corresponding figures were 8.4% and 3.9%, respectively (*P* trend < 0.001, Fig. 2D).

**Fig. 3:**
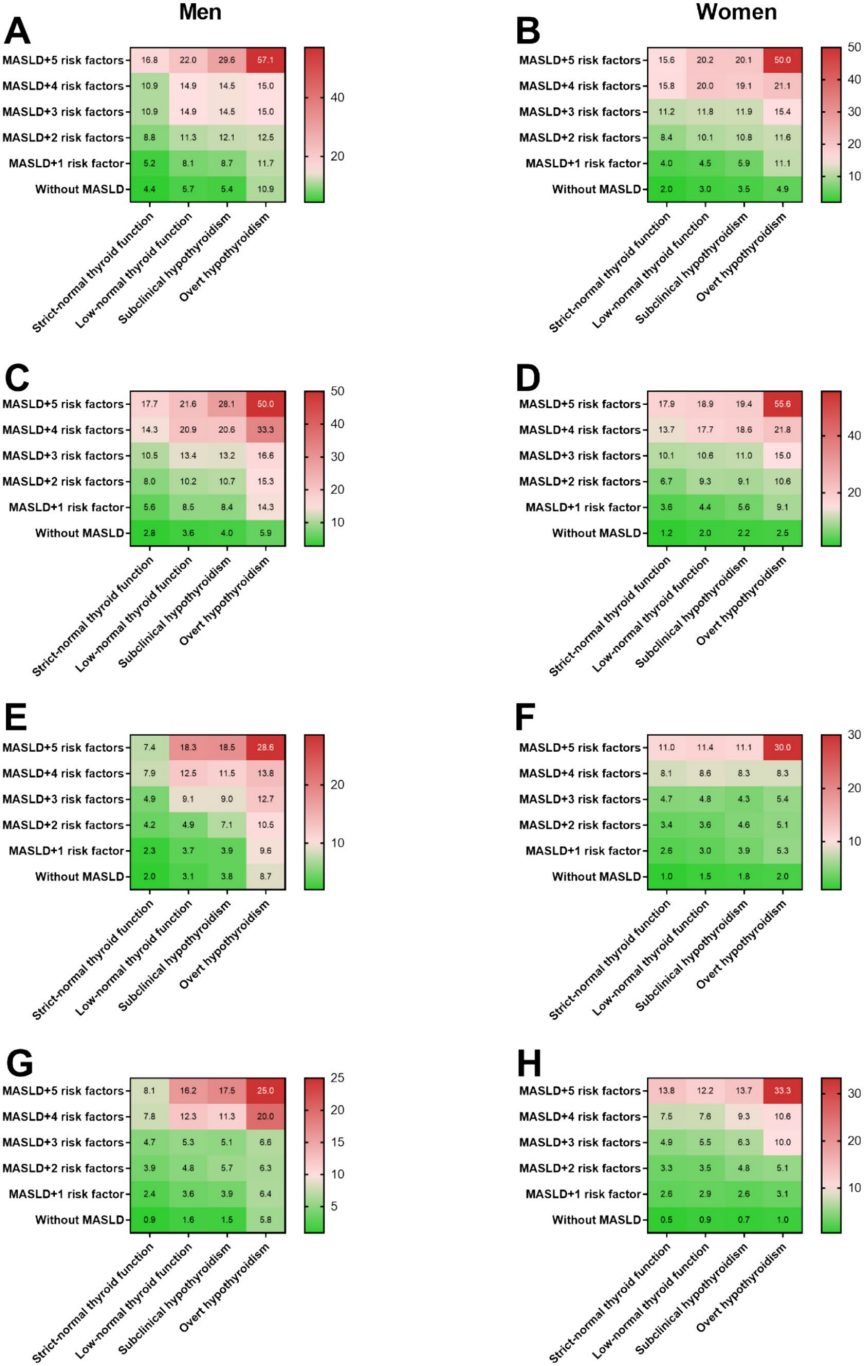
Fibrotic burden across the spectrum of hypothyroidism by metabolic dysfunction-associated steatotic liver disease (MASLD) status and the number of cardiometabolic disorders in men and women. The term “5 risk factors” indicates individuals with five cardiometabolic risk factors that used to define MASLD. **A** Prevalence of significant liver fibrosis in male patients with ultrasonographic-defined MASLD across the spectrum of hypothyroidism by the number of cardiometabolic disorders; **B** Prevalence of significant liver fibrosis in female patients with ultrasonographic-defined MASLD across the spectrum of hypothyroidism by the number of cardiometabolic disorders; **C** Prevalence of significant liver fibrosis in male patients with CAP-defined MASLD across the spectrum of hypothyroidism by the number of cardiometabolic disorders; **D** Prevalence of significant liver fibrosis in female patients with CAP-defined MASLD across the spectrum of hypothyroidism by the number of cardiometabolic disorders; **E** Prevalence of advanced liver fibrosis in male patients with ultrasonographic-defined MASLD across the spectrum of hypothyroidism by the number of cardiometabolic disorders; **F** Prevalence of advanced liver fibrosis in female patients with ultrasonographic-defined MASLD across the spectrum of hypothyroidism by the number of cardiometabolic disorders; **G** Prevalence of advanced liver fibrosis in male patients with CAP-defined MASLD across the spectrum of hypothyroidism by the number of cardiometabolic disorders; **H** Prevalence of advanced liver fibrosis in female patients with CAP-defined MASLD across the spectrum of hypothyroidism by the number of cardiometabolic disorders

Since it is common for an individual to have multiple cardiometabolic disorders and each additional cardiometabolic disorder increases the risk of fibrosis progression [11], we further investigated the effect of the number of cardiometabolic disorders on fibrotic burden across the spectrum of hypothyroidism. As shown in Fig. 3A–H, among both genders, for a given thyroid function, for both the MASLD definition, the proportions of significant and advanced fibrosis increased progressively from individuals having one cardiometabolic disorder to individuals having two, three, four, and five cardiometabolic disorders (all *P* trend < 0.001). For example, significant liver fibrosis in male patients with subclinical hypothyroidism was present in 8.4%, 10.7%, 13.2%, 20.6%, and 28.1% of MASLD (CAP definition) patients having one, two, three, four, and five cardiometabolic disorders (Fig. 3C). The corresponding figures in women were 5.6%, 9.1%, 11%, 18.6%, and 19.4% (Fig. 3D), respectively. Both the proportions of significant and advanced fibrosis were the highest in patients with overt hypothyroidism and five cardiometabolic disorders among both genders.

**Fig. 4:**
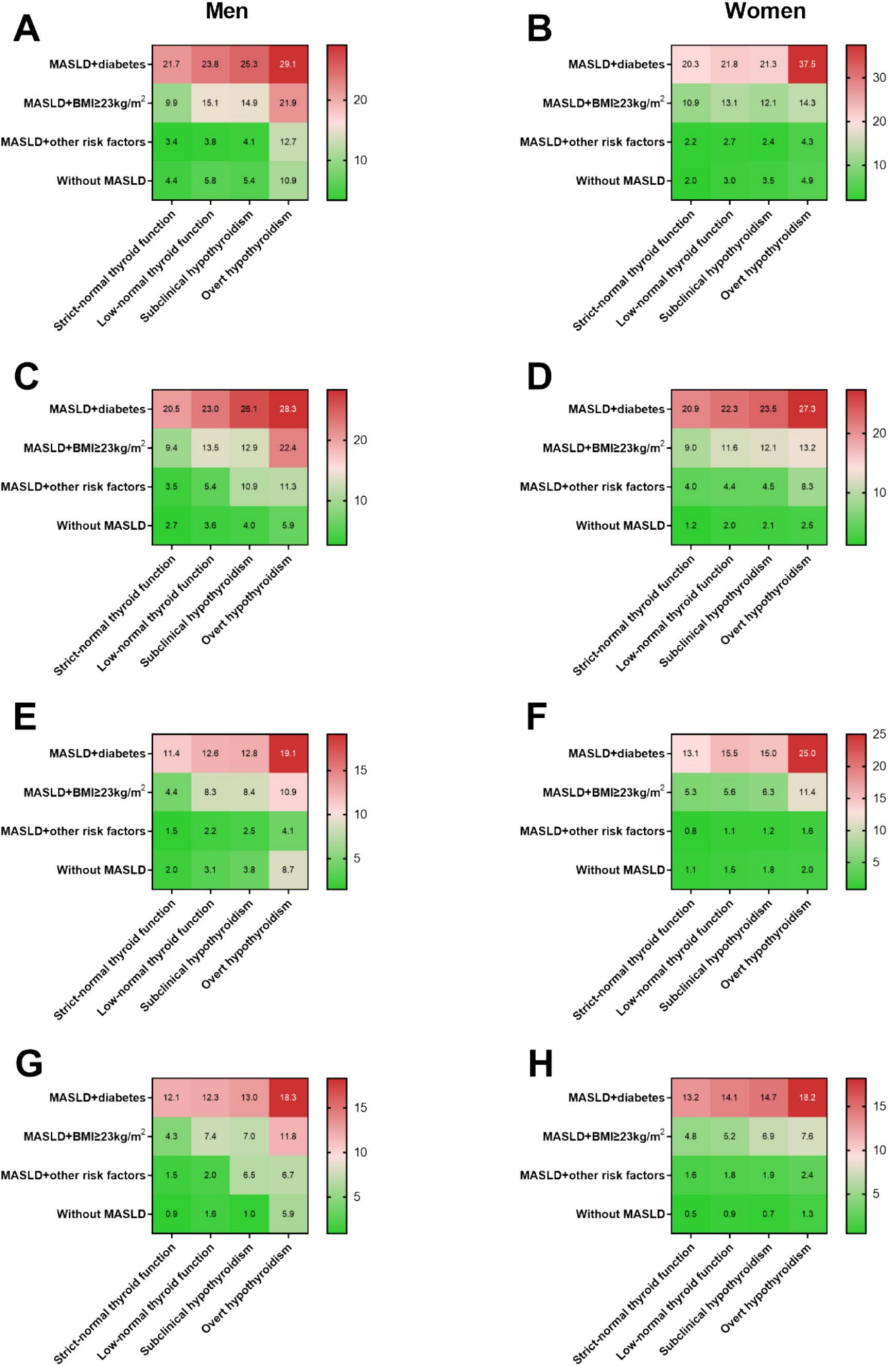
Fibrotic burden across the spectrum of hypothyroidism by metabolic dysfunction-associated steatotic liver disease (MASLD) status and cardiometabolic traits in men and women. The term “other risk factors” indicates MASLD individuals with cardiometabolic risk factors that used to define MASLD in the absence of diabetes and overweight/obesity. **A** Prevalence of significant liver fibrosis in ultrasonographic-defined MASLD across the spectrum of hypothyroidism by cardiometabolic traits in men; **B** Prevalence of significant liver fibrosis in ultrasonographic-defined MASLD across the spectrum of hypothyroidism by cardiometabolic traits in men in women; **C** Prevalence of significant liver fibrosis in CAP-defined MASLD across the spectrum of hypothyroidism by cardiometabolic traits in men; **D** Prevalence of significant liver fibrosis in CAP-defined MASLD across the spectrum of hypothyroidism by cardiometabolic traits in women; **E** Prevalence of advanced liver fibrosis in ultrasonographic-defined MASLD across the spectrum of hypothyroidism by cardiometabolic traits in men; **F** Prevalence of advanced liver fibrosis in ultrasonographic-defined MASLD across the spectrum of hypothyroidism by cardiometabolic traits in women; **G** Prevalence of advanced liver fibrosis in CAP-defined MASLD across the spectrum of hypothyroidism by cardiometabolic traits in men; **H** Prevalence of advanced liver fibrosis in CAP-defined MASLD across the spectrum of hypothyroidism by cardiometabolic traits in women

Because hypothyroidism often coexisted with diabetes and obesity and these two factors outperformed other cardiometabolic disorders in driving fibrosis progression [11], among both men and women, we additionally divided MASLD individuals into three subgroups in the following manner. First, we determined MASLD + diabetes subgroup based on the presence of diabetes (Group 1). Next, in the absence of diabetes, we determined MASLD + BMI ≥ 23 kg/m^2^ subgroup based on the presence of BMI ≥ 23 kg/m^2^ (Group 2). Last, the rest of MASLD patients were categorized as MASLD + other cardiometabolic disorders (Group 3). Among both genders, both significant fibrosis (Fig. 4A–D) and advanced fibrosis (Fig. 4E–H) increased progressively with increasing hypothyroidism grade in patients with each MASLD subgroup, regardless of the definition (*P* trend < 0.001). For example, in men, advanced fibrosis in group 1 (MASLD was defined by CAP value) was present in 12.1% of participants with strict-optimal thyroid function and increased in prevalence in those with low-normal thyroid function (12.3%), subclinical hypothyroidism (13%), and overt hypothyroidism (18.3%) (*P* trend < 0.001) (Fig. 4G). Moreover, for a given thyroid function, for both the MASLD definition, the proportions of significant and advanced fibrosis were the highest in group 1, followed by group 2, and group 3 (*P* trend < 0.001) (Fig. 4A–H). For example, in men, advanced fibrosis in patients with subclinical hypothyroidism was present in 13%, 7%, and 6.5% of participants in group 1, group 2, and group 3 (MASLD was defined by CAP value), respectively (*P* trend < 0.001) (Fig. 4G).

Because hypothyroidism correlates strongly with dyslipidemia and it can promote fibrosis progression [11, 17], we also stratified MASLD individuals by dyslipidemia status. Both significant and advanced liver fibrosis were more present in low-normal thyroid function group, subclinical hypothyroidism group, and overt hypothyroidism group than in the strict-normal thyroid function group (all *P* for trend < 0.001), regardless of dyslipidemia status (Supplementary Table 2). Individuals with hypertriglyceridemia or with decreased HDL-C levels have a greater fibrotic burden than their counterparts across the spectrum of hypothyroidism.

We found that ALT levels were strongly associated with hypothyroidism status (Supplementary Fig. 1). We hence stratified our analysis by ALT levels. Both significant and advanced liver fibrosis increased across the spectrum of hypothyroidism (all *P* for trend < 0.001), regardless of ALT levels (Supplementary Table 3). Individuals with elevated ALT levels have a greater fibrotic burden than their counterparts across the spectrum of hypothyroidism.

Cholestasis might have effects on LSM values. To avoid this effect, we repeated our analysis after excluding patients with abnormal bilirubin (including total and direct bilirubin), results were essentially the same (Supplementary Table 4). To dispel the impact of cholestasis and/or liver congestion, or the potential impact of autoimmune liver disease on the diagnostic accuracy of LSM for fibrosis, we also did sensitive analyses after excluding patients with abnormal bilirubin as well as transaminase levels (including ALT, AST, and GGT). We found that even in subjects with normal bilirubin and transaminase levels, the prevalence of significant and advanced liver fibrosis increased progressively from low-normal thyroid function through subclinical hypothyroidism, to overt hypothyroidism (Supplementary Fig. 2–4).

We then performed a multivariate logistic regression analysis to determine whether thyroid function status was associated with the risk of liver fibrosis. As shown in Table 2, in multivariable analyses, which included demographic and cardiometabolic risk factors, low-normal thyroid function, subclinical hypothyroidism, and overt hypothyroidism in men all increased the risk of significant fibrosis (Table 2), with overt hypothyroidism incurred the highest risk. This was also the case for risk of advanced fibrosis. Women showed a similar pattern. The extent of contributions of the confounding factors were shown in Table 2.

**Table 2:** Associations of hypothyroidism status with hepatic fibrosis in men and women

	Men	Women
Significant liver fibrosis		
Age (years)	1.016 (1.009–1.024)	1.036 (1.019–1.052)
Body mass index (kg/m^2^)	1.002 (1–1.003)	1.001 (0.999–1.004)
Systolic blood pressure	1.019 (1.014–1.023)	1.013 (1.005–1.021)
HbA1c (%)	1.35 (1.263–1.443)	1.282 (1.097–1.497)
Total cholesterol (mmol/L)	0.704 (0.613–0.808)	0.873 (0.647–1.177)
LDL-cholesterol (mmol/L)	1.159 (0.998–1.345)	1.073 (0.772–1.49)
ALT (U/L)	1.018 (1.015–1.021)	1.014 (1.008–1.019)
Total bilirubin (μmol/L)	1.001 (0.987–1.016)	0.961 (0.926–0.998)
MASLD (yes vs. no)	2.154 (1.809–2.564)	2.939 (2.174–3.972)
Anti-diabetic drugs (yes vs. no)	0.91 (0.72–1.096)	0.908 (0.816–1.193)
Lipid-lowering drugs (yes vs. no)	0.84 (0.716–1.198)	0.861 (0.722–1.572)
Blood pressure-lowering drugs (yes vs. no)	0.732 (0.62–1.113)	0.762 (0.646–1.593)
Strict-normal thyroid function	1	1
Low-normal thyroid function	1.472 (1.244–1.743)	1.384 (1.005–1.906)
Subclinical hypothyroidism	1.31 (1.179–1.753)	1.237 (1.198–1.919)
Overt hypothyroidism	1.554 (1.246–3.235)	2.714 (1.417–5.195)
Advanced liver fibrosis		
Age (years)	1.015 (1.004–1.025)	1.042 (1.019–1.064)
Body mass index (kg/m^2^)	1 (0.999–1.002)	1.001 (0.998–1.004)
Systolic blood pressure	1.021 (1.015–1.028)	1.009 (0.998–1.02)
HbA1c (%)	1.403 (1.295–1.521)	1.403 (1.172–1.679)
Total cholesterol (mmol/L)	0.655 (0.538–0.798)	1.07 (0.761–1.505)
LDL-cholesterol (mmol/L)	1.266 (1.025–1.564)	0.724 (0.494–1.062)
ALT (U/L)	1.015 (1.012–1.019)	1.008 (1.004–1.011)
Total bilirubin (μmol/L)	1.008 (0.989–1.027)	0.964 (0.916–1.014)
MASLD (yes vs. no)	2.065 (1.62–2.631)	2.935 (1.944–4.431)
Anti-diabetic drugs (yes vs. no)	0.921 (0.742–1.166)	0.832 (0.764–1.335)
Lipid-lowering drugs (yes vs. no)	0.833 (0.731–1.283)	0.71 (0.642–1.322)
Blood pressure-lowering drugs (yes vs. no)	0.66 (0.544–1.013)	0.612 (0.536–1.476)
Strict-normal thyroid function	1	1
Low-normal thyroid function	1.572 (1.251–1.975)	1.054 (1.004–1.651)
Subclinical hypothyroidism	1.258 (1.14–1.882)	1.058 (1.007–1.939)
Overt hypothyroidism	1.81 (1.217–4.547)	1.973 (1.18–4.864)

Data are presented as odds ratios (95% confidence intervals)

Logistic regression models were used to estimate the associations (ORs with 95% CIs) between thyroid function status and risk of significant or advanced fibrosis

Models were adjusted for age, body mass index, systolic blood pressure, ALT, total bilirubin, HbA1c, total cholesterol, LDL-cholesterol, the presence of MASLD, anti-diabetic drugs, anti-hypertensive drugs, and lipid-lowering drugs

When participants who taking thyroid medications were included in the analysis, results were essentially the same (data were not shown).

## Discussion

This study, to the best of our knowledge, is the first study to establish the magnitude of liver fibrotic burden assessed by VCTE across the spectrum of hypothyroidism. In both men and women, the prevalence liver fibrotic burden (from significant fibrosis through advanced fibrosis to cirrhosis) were significantly greater in subjects with low-normal thyroid function, subclinical and overt hypothyroidism than in subjects with strict normal thyroid function.

In the present study, we identified significant sex differences in fibrotic burden. Men had a greater fibrotic burden than women across the spectrum of hypothyroidism. These findings may indicate that men had a higher propensity for liver fibrosis in the state of hypothyroidism and thus inducing a greater cardiovascular disease and liver-related outcomes.

We noted in our study that even in euthyroid subjects, significant and advanced hepatic fibrosis are increasingly more frequent in the low-normal thyroid function group than in strict normal thyroid function group; Further, in MASLD patients, the low-normal thyroid function group has the similar liver fibrotic burden to the subclinical hypothyroidism group. The highest prevalence of fibrosis was noted in the overt hypothyroidism group. The liver has receptors for TSH. TSH promotes hepatic triglyceride accumulation and hypercholesterolemia by lowering the activity of hepatic lipoprotein lipase, activating the PPAR pathway, the sterol regulatory element-binding transcription factor 1c (SREBP1), and decreasing the phosphorylation of 3-hydroxy3-methylglutaryl coenzyme A reductase [18, 19]. The liver also receives signals from thyroid hormones, and is a major conversion site of fT4 by iodothyronine deiodinase 1 (DIO1) to the metabolically active free triiodothyronine (FT3). FT3 binds its nuclear thyroid receptor β, which located in the liver, to alter transcription of carnitine palmitoyltransferase I (CPT1), which regulates mitochondrial fatty acid oxidation, and SREBP1, which is the master regulator of hepatic de novo lipogenesis. Reduced fT3 and fT4 lead to hepatic triglyceride accumulation by downregulating CPT1, upregulating SREBP1, and lowering activities of hepatic lipases [19]. Hence, in patients with overt hypothyroidism, the concurrent increased TSH level and decreased fT4 or fT3 levels would lead to more accumulation of lipotoxic species, which can promote the fibrosis progression. This may explain why the overt hypothyroidism group had the highest fibrosis cases. Studies showed that even in euthyroid individuals (defined as a TSH level in the upper half of the ‘normal’ range), advanced liver disease has been associated with increased conversion of fT4 to inactive metabolite-reverse-triiodothyronine (rT3) in the liver, at the expense of DIO1-mediated conversion to the active metabolite-fT3 [19–21]. Hence, the reduced intrahepatic thyroid hormone signaling would decrease hepatic conversion of fT4 to fT3 and increase rT3 levels, leading to the accumulation of lipotoxic species. This may explain why there are cases of fibrosis despite the fact that the low-normal thyroid function group is inherently normal in function.

Our findings that significant and advanced fibrosis were present in a non-negligible number of patients with low-normal thyroid function, subclinical and overt hypothyroidism support the notion that hypothyroidism increases the risk of liver fibrosis. Fortunately, emerging evidence showed the therapeutic potential of thyroid hormone receptor β-specific agonists in the reduction of hepatic fat, and fibrosis markers [22, 23]. Significant or advanced fibrosis is closely associated with future risk of cirrhosis, hepatocellular carcinoma, and liver-related and cardiovascular mortality [8]. Therefore, the adverse effect of low thyroid function and its clinical consequences cannot be ignored. The high prevalence of significant and advanced fibrosis across the spectrum of hypothyroidism noted in the present study necessitating a call to action by all clinicians to screen for hepatic fibrosis in patients with hypothyroidism, despite that currently no guidelines recommended this issue.

Our results also provide the first data on effects of cardiometabolic traits on fibrotic burden in MASLD across the spectrum of hypothyroidism. We found that, for a given hypothyroidism grade, liver fibrotic burden differed significantly by cardiometabolic traits, with the prevalence of significant and advanced fibrosis being higher in patients having more cardiometabolic disorders. Knowledge on the interplay of cardiometabolic disorders and hypothyroidism on the prevalence of liver fibrotic burden would be useful for better risk stratification and risk modification, as cardiometabolic traits are readily identifiable and might serve as important targets for secondary prevention to prevent or delay the progression of MASLD to fibrosis. Additionally, hypothyroidism showed additive effects over cardiometabolic disorders on fibrosis risk. Our results also reassure that diabetes is an important driver of hepatic fibrosis in MASLD [24]. Hence, more attention in surveillance and evaluation of liver fibrosis is required in hypothyroidism patients who presented with more cardiometabolic disorders or with diabetes.

It is well-known that cholestasis and/or liver congestion affect the diagnostic accuracy of LSM on fibrosis [25]. Further, reports showed that hypothyroidism exhibits a higher rate of autoimmune liver disease, such as autoimmune hepatitis, primary biliary cholangitis, and primary sclerosing cholangitis [26, 27]; the concurrent autoimmune liver diseases can further promote MASLD to the development of fibrosis in the presence of hypothyroidism. Although autoimmune liver diseases are far less prevalent conditions (the reported incidence is 1–2 per 100,000 population per year for each individual disease) [28], as well as that we excluded patients with liver disease other than MASLD, we cannot neglect the potential possibility that the LSM values can be affected by the undiagnosed or undetected autoimmune liver diseases. Cholestasis liver congestion, and/or autoimmune liver diseases always induce increased transaminase and/or bilirubin levels. To dispel such concerns, we did sensitive analyses after excluding patients with abnormal transaminase levels as well as bilirubin. We found that even in subjects with normal bilirubin and transaminase levels, higher prevalences of significant and advanced liver fibrosis were noted in low-normal thyroid function group, subclinical, and overt hypothyroidism group than in strict-normal thyroid function group. We also assessed the impact of thyroid function status on liver fibrosis aside from cholestasis and liver congestion by adjusting the effect of bilirubin and transaminase levels in logistic regression models. We found that even after adjusting for the effect of bilirubin and transaminase levels, significant and advanced liver fibrosis were significantly associated with low-normal thyroid function, subclinical hypothyroidism, and overt hypothyroidism. The significant and independent associations of thyroid function status with liver fibrosis in the entire study population as well as in the subgroup with normal bilirubin and transaminase levels in our study highlight that thyroid function status, independent of autoimmune liver diseases, cholestasis and liver congestion, predisposes subjects to an increased liver fibrosis risk.

The strength of the present study lies in its largest study population to date. It is the first study to our knowledge to systematically evaluate the prevalence of MASLD and liver fibrotic burden assessed by VCTE across the spectrum of hypothyroidism in an unselected population. Our cohort included apparently healthy individuals who presented for a health check-up, and thus the cohort are believed to be representative of the general population.

The study has several limitations. First, in the absence of liver biopsy to identify hepatic steatosis and fibrosis, misclassification may have occurred. However, this gold standard method is not readily used because of its invasiveness, cost, as well as interpretation challenges (for instance, variability of location of the liver biopsy, histological reading based on sample size, etc.). Second, a causal relationship between the spectrum of hypothyroidism and fibrosis could not be determined due to the cross-sectional study design. Third, our study included only Chinese participants, so the conclusions may not be generalizable to other ethnic groups. Further studies are needed to validate the results of this study for other ethnic/racial groups.

## Conclusions

We found a high prevalence of hepatic fibrotic burden in Chinese adults with overt hypothyroidism and showed that significant and advanced hepatic fibrosis increased in prevalence even in patients without overt hypothyroidism, from individuals with low-normal thyroid function to with subclinical hypothyroidism. Our study also showed a significant difference in liver fibrotic burden according to cardiometabolic traits across the spectrum of hypothyroidism.

## Abbreviations

NAFLD: Non-alcoholic fatty liver disease
TSH: Thyroid stimulating hormone
MASLD: Metabolic dysfunction-associated steatotic liver disease
VCTE: Vibration-controlled transient elastography
LSM: Liver stiffness measurement BP: blood pressure
AUDIT: Alcohol use disorders identification test
FPG: Fasting plasma glucose
TC: Total cholesterol
TG: Triglycerides
LDL-C: Low-density lipoprotein cholesterol
HDL-C: High-density lipoprotein cholesterol
ALT: Alanine aminotransferase
AST: Aspartate aminotransferase
hs-CRP: High-sensitivity C-reactive protein
fT3: Free triiodothyronine
fT4: Free thyroxine
HOMA-IR: Homeostasis model assessment of insulin resistance
CAP: Controlled attenuation parameter
T2DM: Type 2 diabetes mellitus
